# The Role of the Policy Process on Health Service Reconfigurations: Evidence, Path Dependency and Framing: Comment on "‘Attending to History’ in Major System Change in Healthcare in England: Specialist Cancer Surgery Service Reconfiguration"

**DOI:** 10.34172/ijhpm.2022.7642

**Published:** 2022-11-20

**Authors:** Juan I. Baeza

**Affiliations:** King’s College London, London, UK

**Keywords:** Service Reconfiguration, Policy Process, Evidence, Path Dependency, Framing

## Abstract

Historically healthcare services have largely developed on an incremental basis, with various piecemeal changes and some notable policy leaps that illustrate a punctuated equilibrium health policy process. More recently policy-makers have attempted, successfully and unsuccessfully, to reconfigure healthcare services to address perceived problems in the delivery of important services such as stroke, cancer, and trauma. Perry et al provide a welcome addition to research in this area by focusing on the importance of history in a reconfiguration of cancer services in Greater Manchester (GM). Perry et al analyse how and why this configuration was successful after several failed attempts in the past and in this commentary, I want to reflect on the explanatory role health policy analysis can contribute to studying the reconfiguration of healthcare services.

 Fraser et al^1^ highlight a growing international literature on various aspects of health system reconfigurations that have appeared in the past two decades and Perry and colleagues’^2^ paper is a welcome contribution to this blossoming field. The various studies thus far have illustrated that reconfiguring healthcare services is characterised as difficult, contentious, and conflictual. Perry and colleagues’ paper focuses on how history influences these reconfiguration attempts, adding an important ingredient to this growing evidence base. However, it is worth noting that much of their data rely on individuals recalling events that occurred more than 17 years ago, which has obvious limitations. Perry et al use Best and colleagues’^3^ definition of what they refer to as major system change (MSC) as, “coordinated, system-wide change affecting multiple organisations and care providers, with the goal of making significant improvements in efficiency of healthcare delivery, the quality of patient care, and population-level patient outcomes” (p. 422). Their study focuses on the reconfiguration of specialist cancer surgery services in Greater Manchester (GM).

 They highlight how power in general and the power dynamics amongst the various stakeholders in particular are crucial elements to consider in the MSC process. Other reconfiguration studies have also considered the issue of history, Fraser and colleagues’ study charted the interplay between evidence, power, and policy, within a historical context. MSC can be examined as a policy process and within this process history plays a significant role. The policy process is a broad and complex topic and a commentary has to be necessarily selective and for this reason this paper will be examining three key aspects of the process that could be usefully considered in MSC analysis and that intersect with the concept of ‘history’ in Perry and colleagues’ paper. The three features this commentary focuses on are evidence, path dependency (PD) and framing.

 An important backbone to recent healthcare reconfigurations is the use of evidence to support the need for the reconfiguration of certain healthcare services, be they stroke, trauma or as in this case cancer services. The evidence used in these instances seeks to empirically illustrate how a reconfigured service would provide improved patient outcomes. The debate about service reconfiguration is prompted by a widespread perception and general agreement that *something* must be done, such agreement would ordinarily be a promising foundation for reforming services. Indeed, at the start of a reconfiguration journey most stakeholders agree that some kind of reconfiguration of services is needed, what is not agreed upon is what the new configuration should look like. In policy analysis terms, the problem stream is largely agreed upon but the policy and politics streams are contested.^4^ The literature on MSC illustrates that it is not enough to be able to merely provide robust evidence of improved patient outcomes to justify a reconfiguration, how the evidence is marshalled and presented is equally, if not more important. There are also limitations to the use of evidence, they may indicate how many specialist centres are needed but where these should be located can be and often is contested by the various stakeholders. In this context we could usefully add *where* to Lasswell’s definition of politics, “Who gets what, when and how.”^5^ Perry et al clearly highlight how various historical reviews and reports into GM cancer services pointed to the need for service reconfiguration and a reduction in hospitals undertaking cancer surgery to improve patient outcomes, but evidence alone was not enough to assure success, in GM it took 13 years from presenting evidence through reports and reviews that a reconfiguration was needed to the new reconfigured service being finally launched. This would suggest that Kingdon’s multiple streams framework of problem, policy and politics all need to be aligned before action occurs, and this often takes time.^4^

 This points to the importance of PD, a key aspect of historical institutionalism^6^ and links well to Perry and colleagues’ focus on history. PD helps explain why we are where we are, history matters, and history endures over time and is important in shaping future decisions. However, it is not enough to just acknowledge that history matters, if policy-makers are to make progress, they need to understand *how* history matters and that different stakeholders will have different versions of history. PD helps to explain the persistence and endurance of institutions and it is cherished institutions in the shape of hospitals that are often at risk in any reconfiguration exercise, understanding and attending to this is crucial in any reconfiguration process. Understanding how history matters requires local knowledge, which is likely to vary from place to place and is why the wide-ranging place-based literature on healthcare reconfigurations that we now have is so useful and important. In addition, it would also have been helpful for Perry et al to have set their paper within the much longer historical and rich research into GM health services that exists.^7^

 Policy framing is the third crucial aspect of the healthcare policy process, it influences evidence and PD by shaping how these are perceived by the various stakeholders.^8^ Framing and agenda setting are important topics in relation to studying how the media influence public opinion, similarly, they are crucial factors when one is analysing health policy.^9^ The reconfiguration literature highlights the complex and varied ‘audience’ that is involved in the reconfiguration policy process. The audience is made up of politicians, policy-makers, managers, health professionals and the public and it is important to note that none of these groupings are internally homogenous or clearly distinct. If policy-makers do not take care of how to frame the debate early in the process, other groups will, simply producing a review with robust evidence is not enough as countless health service reviews illustrate.^10^

 Evidence is not neutral; its framing will strongly influence its impact. Similarly, the past is not something that is agreed upon by everyone and how history is framed will also influence what might be acceptable in the future. The framing of a process needs to be established very early in the policy process, as this will significantly determine what issues are considered, what aspects are in and out of the ‘frame,’ in other words what is to be considered ‘important’? Defining the ‘problem’ within a particular frame shapes the diagnosis, which itself leads to a particular conclusion.^11^ Framing an issue enables one to select and highlight certain aspects of a perceived reality and make them more salient to promote a particular way of defining the problem that promotes a causal theory to this ‘problem,’ which logically leads to a particular recommendation. As Edelman^12^ elegantly states, “The social world is a kaleidoscope of potential realities, which can be readily evoked by altering the ways in which observations are framed.”

 All these policy process factors must be viewed through the prism of power. In their paper, Fraser et al clearly point out that there are various manifestations of power, some are structural, such as hierarchical and jurisdictional power and others are post-structural, for example, language and discourse. Structural power influences how policy-makers undertake and develop policy, Alford’s structural interest groups approach is often used in health policy analysis.^13^ A vivid example of this would be the creation of the British National Health Service (NHS) in 1948, the then Minister of Health, Anuerin Bevan, recognised that the most structurally powerful interest group involved in this policy initiative were the hospital consultants and he famously, “stuffed their mouths with gold” to support his policy of socialised medicine.^14^ However, the power of language and discourse also needs to be considered in the policy process. Fraser et al usefully link discursive power with framing by suggesting, “…that the discursive mobilisation of clinical research evidence to frame hospital reconfigurations in recent years is an increasingly important technique….” They go on to assert that any proposed change in services that can be framed as ‘evidence based’ has great rhetorical power, giving its proponents legitimacy.^1^

 The contemporary health policy landscape that any service reconfiguration is to be carried out in needs careful consideration, and this landscape will have been shaped by past policy decisions. Healthcare service reconfigurations are essentially the result of health service planning, which is the opposite to the unpredictable and unplanned results of competition, which has been an important plank of NHS policy for the past 30 years. Perry et al and other reconfiguration literature suggest that it is challenging to promote a rationally planned service into an English health service landscape that has encouraged competition through an internal market and the institutional independence of hospitals through the promotion of Foundation Trusts. It is not only local history that is significant, but the national health policy history is also important and can act, in this case, as a PD block to change.

 Finally, the paper by Perry and colleagues is also important by highlighting the type of reconfiguration research that is needed. As we have already discussed reconfigurations take time, are unpredictable and involve a variety of stakeholders. For these reasons it is important for research into this topic to be extensive, ongoing and fine grained, Perry’s team should be congratulated for their rich, broad, three years long data collection, which has resulted in a valuable addition to the growing literature into health service reconfigurations.

## Ethical issues

 Not applicable.

## Competing interests

 Author declares that he has no competing interests.

## Author’s contribution

 JIB is the single author of the paper.
